# Exercise-Induced Troponin Elevation in High-Performance Cross-Country Skiers

**DOI:** 10.3390/jcm13082335

**Published:** 2024-04-18

**Authors:** Tom Kastner, Florian Frohberg, Judith Hesse, Bernd Wolfarth, Jan C. Wuestenfeld

**Affiliations:** 1 Institute for Applied Training Science, Marschnerstrasse 29, 04109 Leipzig, Germany; kastner@iat.uni-leipzig.de (T.K.); judith.a.hesse@googlemail.com (J.H.); bernd.wolfarth@charite.de (B.W.); 2 Department of Sports Medicine, Universität zu Berlin, Charitéplatz 1, 10117 Berlin, Germany

**Keywords:** troponin I, troponin T, sport, elite athletes, exercise, cardiac health, cross-country skiing

## Abstract

**Background:** Troponin I and T are biomarkers to diagnose myocardial infarction and damage. Studies indicate that strenuous physical activity can cause transient increases in these troponin levels, typically considered physiological. However, current data show differences in the exercise-induced increase in troponin I and T in elite athletes. **Method:** This prospective clinical study aimed to determine troponin I and T levels in 36 top cross-country skiers of the German national team (18 male, 18 female) after a standardized competition load over two days. All study participants underwent a comprehensive sports medical and cardiological evaluation, including ECG and echocardiography. A multivariable regression analysis was utilized to identify possible predictors of increased troponin I levels. **Results:** Only three male athletes (8.1%) showed an isolated increase in Troponin I (Ø 112.49 ng/L, cut off < 45.2 ng/L), while no increase in troponin T in the study population was detected. **Conclusions:** The analysis suggested several potential predictors for increased troponin I levels, such as height, weight, weekly training hours, and indications of an enlarged sports heart, though none achieved statistical significance. Knowing the different exercise-induced detectability of the various troponins in the clinical setting is essential.

## 1. Introduction

The increase in troponin caused by physical exertion is a well-known phenomenon described in the literature [1]. Multiple studies have consistently reported these abnormalities since the early to mid-2000s [2,3,4,5]. Although a physiological mechanism of transitory troponin release is predominantly assumed, there is also some indication of a possible pathological genesis [6,7,8]. Troponin tests have been available since the 1980s, but it was not until the mid-1990s that they were introduced into clinical practice in the USA and Germany. Since then, they have undergone continuous development [9,10]. Troponin, a cardiac biomarker, is released when heart muscle cells are damaged and is a crucial tool in diagnosing acute myocardial damage, including acute myocardial infarction [11,12]. Troponin is essential to routine and emergency laboratory and cardiology diagnostics due to its high sensitivity. In addition, cardiac troponin is also considered a general marker for damage to the heart muscle, and data have indicated the prognostic relevance of elevated troponin levels for subclinical cardiovascular dysfunction [13]. If troponin values are elevated after physical exertion, it is therefore recommended to check these values (regardless of whether troponin T or I is used) to rule out myocardial damage. The general clinical assessment should also be taken into account [14]. Research has indicated a physiological increase in cardiac activity in healthy athletes [9,15]. Studies have demonstrated that temporary increases in troponin levels can occur during intense physical activity, such as marathon running, typically over 24 to 72 h [14,15]. Aengevaeren et al. [16] conducted a study that revealed temporary increases in cardiac troponin I levels in 7 out of 11 participants in a marathon race. Furthermore, 11 out of 11 subjects exceeded the upper reference limit for cardiac troponin T, providing strong evidence for exercise-induced troponin elevations. However, despite similar release kinetics of troponin I and troponin T, the values after exercise did not correlate significantly. These data once again raise the question of the underlying physiological mechanisms. While most studies on exercise-induced troponin elevation have focused on amateurs and recreational athletes, typically after endurance competitions, there is limited knowledge on troponin release in elite athletes. Wuestenfeld et al. [17] found that a relevant proportion in a large group of top athletes, primarily from endurance sports, showed increased troponin I levels, with considerable differences in detectability between troponin I and T. However, it is essential to note that the study did not record individual preloads in the context of daily training load. The present study investigated the frequency of troponin increase in a homogeneous group of top athletes in a single endurance sport in the context of daily training load and after a structured competition load. Possible predictive parameters of anthropometric, electrocardiographic, and echocardiographic examinations that indicate increased troponin elevations were analyzed.

## 2. Materials and Methods

### 2.1. Participants

Thirty-six high-performance cross-country skiers (18 female, 18 male) with a median age of 22.0 years (IQR 5.0 years), all German national cross-country skiing team members, were included in this prospective clinical study. A further description of the study population is shown in Table 1. A venous blood sample was taken as part of the standard medical care for high-performance athletes in September 2020 in the morning, 20 h after a standardized competition load over two days: a running competition the day before with a distance of 5 km for the women and 10 km for the men. Two days earlier, a ski sprint competition took place over a distance of 1.1 km (to be run four times by each athlete).

### 2.2. Troponin Tests

The quantitative measurement of high-sensitivity cardiac troponin T (hs-cTnT) from the venous blood samples was performed using the cobas Elecsys Troponin T hs STAT assay (Roche Diagnostics GmbH, Mannheim, Germany). The cutoff level for myocardial damage is 14 ng/L. For the quantitative measurement of serum high-sensitivity troponin I (hs-cTnI), the Atellica IM Analyzer system (Siemens Healthineers AG, Erlangen, Germany) was used together with the High-Sensitivity Troponin I (TnIH) assay. This assay’s cutoff level for myocardial damage is 45.2 ng/L. 

### 2.3. Medical Examination

All athletes underwent a complete sports medicine and cardiology examination three months beforehand. In addition to a medical history and clinical examination, this included electrocardiography and echocardiography with the parameters absolute cardiac volume, relative cardiac volume (as described by the equation of Dickhut 1996 [18]), septal thickness in diastole, diastolic dimensions of the left ventricle and right ventricle and diastolic diameter of the left atrium. Furthermore, a detailed anthropometric examination of the athletes was carried out with the parameters of body height, body weight, body fat percentage and lean body mass. 

### 2.4. Ethical Considerations

This study was conducted in strict compliance with the ethical principles set out in the Declaration of Helsinki. The blood sample was taken by medical staff as part of regular standardized medical testing during the season. For the determination of the cardiac enzymes troponin I and T from this standard blood sample, informational materials and verbal information were provided in advance. Written consent was obtained from the athletes for the scientific use of the data in anonymized form. All athletes were healthy at the time of sampling and showed no symptoms of infectious disease. In the preceding sports medical examination, in accordance with the guidelines of the German Society for Sports Medicine and Prevention (DGSP), internal diseases, in particular of the cardiovascular system, were excluded.

### 2.5. Statistical Analysis

The statistical analyses aimed to determine correlations between the biomarker troponin I and anthropometric, electrocardiographic, and echocardiographic parameters. The analysis was conducted using SPSS Statistics Version 28 (IBM Corp., Armonk, NY, USA).

Descriptive statistics were presented as median and interquartile range (IQR) to provide robust measures of central tendency and variability, especially suitable for data that may have outliers, or not be normally distributed. Special attention was given to the distribution of quantitative variables due to the small size of the study group. The Shapiro-Wilk test was utilized to evaluate the normality of the data distribution, which is particularly appropriate for small sample sizes and offers a reliable assessment of whether the data adhere to a normal distribution. Upon assessing normality, correlation analyses were undertaken to identify potential linear relationships between troponin I and other variables. Pearson’s correlation coefficient was calculated when variables demonstrated normal distribution, while Spearman’s rank correlation was employed for variables which were not normally distributed. The selection of correlation test was based on the distribution characteristics of the data to ensure the most accurate method was applied for determining the strength and direction of the relationships. Subsequent to the correlation analysis, a multivariable regression analysis was conducted. Variables incorporated into the model—height, weight, lean body mass, training hours per week, absolute heart volume, interventricular septal thickness in diastole, and diameter of the left atrium—were selected based on their preliminary correlation with troponin I. The adequacy of the regression model was evaluated using the coefficient of determination (R^2^) and adjusted R^2^, which indicated the proportion of variance in troponin I levels explained by the collective independent variables. F-tests were performed to determine the overall significance of the model, while *t*-tests for each regression coefficient were conducted to assess the significance of each independent variable’s contribution to the model.

## 3. Results

This study analyzed 36 high-performance cross-country skiers, all members of the German national cross-country skiing team, with a median age of 22.0 years (female: 24.0 years, male: 22.0 years). The measurement data of the study population are listed in Table 2, Table 3 and Table 4. 

All athletes passed their medical examinations with no clinical or pathological findings detected in their internal or cardiovascular systems. Most athletes showed typical sports physiological changes in their resting ECG and echocardiographic examination, including prolongation of the QRS duration with signs of incomplete right bundle branch block, sinus bradycardia, and harmonic sports heart enlargement with an increase in the muscular wall thickness and internal diameter of all heart cavities. It is important to note that left ventricular function was normal in all athletes, with a left ventricular ejection fraction > 60%.

### 3.1. Troponin Values

None of the athletes had a troponin T value above the 14 ng/L reference range. In contrast, three male athletes (8.1% of the study population) had troponin I values above the 45.2 ng/L cut-off. The median value was 127.4 ng/L, ranging from 54.0 to 155.9 ng/L. These three athletes were all assigned to the perspective squad (Germany’s second-highest squad development level) and had a steep type in the ECG. In the group of athletes (n = 34) with troponin I values within the reference range of 45.2 ng/L, the median troponin value was 8.6 ng/L (IQR = 14.1). The troponin values for the whole study population are shown in Table 5.

### 3.2. Statistical Analysis

The correlation analysis revealed weak to moderate correlations between troponin I and the investigated parameters, although none showed a strong linear relationship (see Table 6).

The statistical analysis incorporated a regression model to assess the influence of several variables on troponin I levels, including height, weight, training hours per week, heart volume absolute, interventricular septal thickness in diastole, and diameter of the left atrium. This analysis yielded an R-squared value of 0.133, signifying that the model accounted for approximately 13.3% of the variability observed in troponin I levels based on these factors. Additionally, the model produced an adjusted R-squared value of −0.046 with an effect size (Cohen’s f^2^) of 0.153. The regression coefficients for each variable were calculated along with their standard errors, t-values and *p*-values, as shown in Table 7. The analysis indicated that none of the independent variables contributed significantly to explaining the variability of troponin I levels within the data set.

## 4. Discussion

Exercise-induced transient increase in cardiac troponin is a well-known phenomenon. However, the underlying causes have not yet been sufficiently clarified, and determining these parameters in athletes can lead to misinterpretations in the context of clinical questions [19]. Furthermore, it is still not completely clear whether there are differences between stress-induced increases in troponin I and troponin T. Wuestenfeld et al. [17] investigated the spontaneous occurrence of increases in troponin I and T values in a group of 219 athletes out of competition. They found that that 11.4% of the athletes had elevated troponin I and/or troponin T values, regardless of the training load prior to sample collection. This indicates that increases in troponin I and T levels occur spontaneously in out-of-competition athletes, but the underlying causes are unclear.

The present study examined troponin I and T levels in a homogeneous group of 36 high-performance cross-country skiers after a defined competition load. The aim was to use a standardized high-intensity preload in a cardiologically well-studied and homogeneous group of athletes to identify correlations that could further clarify the occurrence of exercise-induced troponin elevation. Current anthropometric, electrocardiographic, and echocardiographic data were available for all athletes. Three male athletes (8.1% of the study population) showed troponin I values above the limit range, and no one showed troponin T elevation. This finding is in line with the study of Wuestenfeld et al. [17], where most of the positive troponin elevations were those of troponin I rather than troponin T. Furthermore, the frequency of occurrence of troponin values above the upper range limit is comparable to the study results of Wuestenfeld et al. (8.1% vs. 11.4%), while in other studies, which were mostly conducted on significantly older athletes, a stress-induced troponin increase was usually found much more frequently (in some cases > 50%) [20,21,22,23,24].

The time of sample collection can influence the level of the measured values. Baker et al. [14] analyzed 12 studies in which troponin was determined after prolonged exercise. The trend showed the highest values 0 to 3 h after the end of the exercise, whereby the data was mainly based on troponin T. For troponin I, a concentration peak was observed 4 to 6 h post-exercise [25]. For this reason, an underestimation of the troponin values is possible for the present study, with only one measurement having been taken after 20 h. 

In addition to the absolute analysis of the occurrence of troponin I and T elevations within this group of cardiac healthy cross-country skiers, predictors for elevated troponin I levels were investigated. Correlation analysis revealed weak to moderate relationships between troponin I and the variables analyzed, indicating that while there is some degree of association, none of these variables strongly predict troponin I levels. This suggests that changes in troponin I levels are only slightly associated with changes in the selected variables, without any of them being a robust predictor. The multivariable regression analysis, incorporating variables such as height, weight, weekly training hours, absolute heart volume, interventricular septal thickness in diastole, and left atrial diameter, yielded an R-squared value of 0.133. Additionally, the adjusted R-squared value, which accounts for the number of predictors in the model and the sample size, was negative, indicating that the model’s explanatory power was less than that of a simple mean model, and suggesting overfitting or an inappropriate model for the data complexity. This means that approximately 13.3% of the variability in troponin I levels can be accounted for by these variables, yet the negative adjusted R-squared value signals that the model explains only a small fraction of the changes in troponin I levels and may not be the most suitable for these data. Furthermore, none of the variables included in the regression model emerged as statistically significant predictors of troponin I levels, as evidenced by the high *p*-values associated with each parameter. This indicates that a clear influence of these specific parameters on troponin I levels could not be established based on the data analyzed. The absence of statistically significant findings from both linear and multivariable regression analyses might suggest that the relationships between troponin I and the investigated parameters are either not linear or may be affected by other factors not measured or included in this study. 

Several studies with different conclusions have been published on possible predictors of exercise-induced troponin release. Age, gender, body composition, training intensity, training duration, training age, cardiovascular health (including blood pressure), hydration status, and various environmental factors are among the predictors that have been identified [25]. Exercise intensity and duration have shown the most significant influence on post-exercise troponin values [26,27], although the data situation needs to be clarified. In a meta-analysis with 26 included studies, Shave et al. [28] showed that shorter exercise times can lead to increased troponin T values after exercise. Marshall et al. [29] and Weippert et al. [30] showed that shorter and more intense exercise leads to higher troponin concentrations than longer moderate intensity. In contrast, Lara et al. found more elevated troponin I levels the longer the race distance when comparing a marathon distance with a half marathon distance and a 10 km race [31]. Additionally, Eijsvogels et al. [25] found a significant correlation with exercise duration in post-marathon troponin I determination. Most likely, troponin release depends on the total cardiac workload, which is the product of exercise intensity and duration [25]. The age of the probands is negatively related to troponin release [27,32]. Whether individual training status can influence troponin release has not been conclusively clarified [25]. Due to the homogeneity of the subject collective in our study concerning age, training status, and standardized preload, no conclusions can be drawn. However, since the present study included two high-intensity competition workloads that led to an increase in troponin I above the normal range in only three of thirty-six athletes, it was again possible to show that the phenomenon of exercise-induced troponin elevation is a comparatively rare phenomenon that also occurs in highly endurance-trained young athletes. The measured troponin values underline the statement by Aengvaeren et al. [25] that post-exercise troponin values can vary within an athlete cohort with a similar exercise bout. 

## 5. Conclusions

Almost one-tenth of the athletes showed elevated troponin I values, with simultaneously normal troponin T values. Although long-term effects were not considered, this study provided further evidence of the physiological nature of troponin I values above the normal range in heart-healthy endurance athletes after exercise. Nevertheless, the underlying mechanisms are still not understood. There were indications but no significant correlations found after investigating associations of elevated troponin I values with specific anthropometric, electrocardiographic, and echocardiographic parameters. 

The results of this study have emphasized that cardiac troponins I and T should be differentiated in the context of a clinical question to distinguish between exercise-induced and pathological elevations. The reasons for the differential release of troponin I and T, which are used in clinical practice as equivalent parameters for the detection of myocardial damage, must be analyzed.

Further investigations to shed light on possible causes and correlations of increased troponin release are necessary to ensure a reliable interpretation of this laboratory constellation in protecting the cardiovascular health of athletes. These should include larger sample sizes in the special population of high-performance athletes and, if possible, be conducted as intervention studies with standardized load and monitoring of troponin progression after exercise.

## Figures and Tables

**Table 1:** Baseline data of athletes (values are presented as median and interquartile range [IQR]).

All *n* = 36	Female *n* = 18	Male *n* = 18
Age (years)	24.0	22.0
IQR	7.0	3.3
Olympic squad	2	0
Perspective squad	10	11
Complementary squad	0	2
Junior squad	6	5
Training sessions per week	10.0	11.0
IQR	2.8	2.0
Training hours per week	18.0	20.0
IQR	6.8	6.8

**Table 2:** Baseline anthropometric data of all athletes (values are presented as median and interquartile range [IQR]).

Anthropometric Parameter	All	Female	Male
Height (cm)	178.6	169.0	184.8
IQR	16.1	10.3	7.6
Weight (cm)	68.0	58.4	74.2
IQR	16.1	6.2	7.5
BMI (kg/m^2^)	21.6	20.7	22.0
IQR	2.1	1.9	1.5
Body fat (%) caliper	10.9	12.7	10.1
IQR	3.6	6.1	1.2
Lean body mass (kg)	60.9	51.2	67.2
IQR	16.3	5.6	6.2
Fat mass (kg)	8.9	13.3	6.1
IQR	7.3	3.4	1.3

**Table 3:** Baseline resting ECG data of all athletes (values are presented as median and interquartile range [IQR]).

ECG Parameter	All	Female	Male
Left axis deviation	1	0	1
Right axis deviation	3	2	1
Normal axis	9	4	5
Vertical axis	23	12	11
PQ duration	145.5	142.0	149.5
IQR	27.8	30.5	27.8
QRS duration	107.5	103.0	113.0
IQR	13.5	13.3	8.5
QTc Time (Bazett)	407.5	417.0	398.5
IQR	28.0	30.3	33.5
Resting heart rate	49.0	52.0	48.0
IQR	10.0	15.0	6.0

**Table 4:** Baseline echocardiographic data of all athletes (values are presented as median and interquartile range [IQR]).

Echocardiographic Parameter	All	Female	Male
Heart volume/body weight (mL/kg)	13.2	13.0	13.3
IQR	1.6	2.1	1.3
Diameter interventricular septum (cm)	1.0	0.9	1.1
IQR	0.2	0.2	0.2
Diameter left atrium (cm)	3.7	3.6	3.8
IQR	0.30	0.2	0.3
Diameter left ventricle (cm)	5.1	5.0	5.3
IQR	0.5	0.4	0.4

**Table 5:** Troponin I and troponin T values (values are presented as median and interquartile range [IQR]).

Troponin I and Troponin T (ng/mL)	All	Female	Male
Troponin I	15.2	5.4	13.0
IQR	19.4	27.6	12.5
Min/Max	3.0/155.9	3.0/37.0	4.4/156.0
Troponin T	<14	<14	<14

**Table 6:** Linear correlation analysis between troponin I and the investigated parameters.

Variable	*r*-Value	*p*-Value
Age	0.077	0.656
Training sessions per week	0.158	0.359
Training hours per week	0.240	0.159
Height	0.207	0.226
Weight	0.217	0.205
Body mass index	0.128	0.459
Body fat per calliper	−0.147	0.393
Lean body mass	0.272	0.109
Fat mass	−0.275	0.110
Heart rate rest	−0.374	0.025
PQ duration	0.123	0.477
QRS duration	0.211	0.218
QTc Time (Bazett)	−0.319	0.058
Heart volume absolute	0.310	0.066
Heart volume/body weight	0.218	0.202
Diameter interventricular septum	0.313	0.063
Diameter left atrium	0.276	0.103
Diameter left ventricle	0.059	0.733

**Table 7:** Multivariable regression analysis, limited to the variables of height, weight, lean body mass, training hours, heart volume absolute, interventricular septal thickness in diastole, and diameter of the left atrium.

Variable	Coefficient	Standard Error	t-Value	*p*-Value
Const.	−152.287	218.438	−0.697	0.491
Height	0.425	1.457	0.292	0.772
Weight	−0.253	1.778	−0.142	0.888
Training hours	1.224	1.751	0.699	0.490
Hear volume absolute	0.003	0.122	0.699	0.490
Diameter interventricular septum	37.150	62.280	0.597	0.555
Diameter left atrium	14.390	35.197	0.409	0.686

## Data Availability

All original data from this study are available to the authors in digital form and can be made available on request.
